# 4-Chloro-2-((1*R*)-1-{[(*R*)-(2-chloro­phen­yl)(cyclo­pent­yl)meth­yl]amino}eth­yl)phenol

**DOI:** 10.1107/S1600536808034624

**Published:** 2008-10-25

**Authors:** Guang-You Zhang, Ting Yang, Bao-Wang Xu, Di-Juan Chen, Wan-Hui Wang

**Affiliations:** a School of Chemistry, Jinan University, Jinan 250022, People’s Republic of China; b Qilu Pharmaceutical Co. Ltd, Shandong Province, Shandong 250100, People’s Republic of China; c Graduate School of Science and Engineering, Saitama University, 255 Shimo-ohkubo, Sakura, Saitama 338-8570, Japan

## Abstract

The title compound, C_20_H_23_Cl_2_NO, was prepared by condensation of (*R*)-1-(2-chloro­phen­yl)-1-cyclo­pentyl­methanamine with 1-(5-chloro-2-hydroxy­phen­yl)ethanone, resulting in the formation of a new chiral center. The structural analysis confirms the absolute configuration of the title compound and the formation of the (*R*,*R*) diastereoisomer. There is an intra­molecular O—H⋯N hydrogen bond which stabilizes the conformation of the mol­ecule. The mol­ecules are linked to each other through weak C—H⋯π inter­actions.

## Related literature

For general background, see: Ager *et al.* (1996 ▶); Berrisford *et al.* (1995 ▶); Cimarelli & Palmieri (1998 ▶); Cimarelli *et al.* (2001 ▶, 2002 ▶); Hayase *et al.* (1997 ▶); Nakano *et al.* (1997 ▶); Palmieri (1999 ▶, 2000 ▶); Soai & Niwa (1992 ▶); Watanabe *et al.* (1991 ▶); Xu & Pu (2004 ▶); Yang *et al.* (2005 ▶).
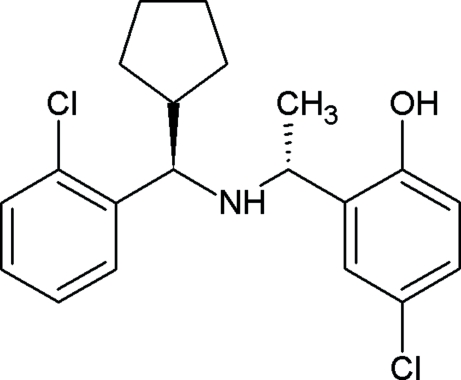


## Experimental

### 

#### Crystal data

C_20_H_23_Cl_2_NO
*M* *_r_* = 364.29
Orthorhombic, 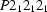

*a* = 11.286 (2) Å
*b* = 11.539 (2) Å
*c* = 14.740 (3) Å
*V* = 1919.5 (6) Å^3^
*Z* = 4
Mo *K*α radiation
μ = 0.34 mm^−1^
*T* = 298 (2) K
0.42 × 0.29 × 0.18 mm

#### Data collection

Bruker SMART CCD area-detector diffractometer
Absorption correction: multi-scan (*SADABS*; Bruker, 1997 ▶) *T*_min_ = 0.869, *T*_max_ = 0.941
10145 measured reflections
3573 independent reflections
2761 reflections with *I* > 2σ(*I*)
*R*_int_ = 0.032

#### Refinement

*R*[*F* ^2^ > 2σ(*F* ^2^)] = 0.048
*wR*(*F* ^2^) = 0.113
*S* = 1.02
3573 reflections
219 parameters
H-atom parameters constrained
Δρ_max_ = 0.17 e Å^−3^
Δρ_min_ = −0.22 e Å^−3^
Absolute structure: Flack (1983 ▶), 1629 Friedel pairs
Flack parameter: 0.03 (8)

### 

Data collection: *SMART* (Bruker, 1997 ▶); cell refinement: *SAINT* (Bruker, 1997 ▶); data reduction: *SAINT*; program(s) used to solve structure: *SHELXS97* (Sheldrick, 2008 ▶); program(s) used to refine structure: *SHELXL97* (Sheldrick, 2008 ▶); molecular graphics: *ORTEPIII* (Burnett & Johnson, 1996 ▶) and *ORTEP-3 for Windows* (Farrugia, 1997 ▶); software used to prepare material for publication: *SHELXL97*.

## Supplementary Material

Crystal structure: contains datablocks I, global. DOI: 10.1107/S1600536808034624/dn2393sup1.cif

Structure factors: contains datablocks I. DOI: 10.1107/S1600536808034624/dn2393Isup2.hkl

Additional supplementary materials:  crystallographic information; 3D view; checkCIF report

## Figures and Tables

**Table 1:** Hydrogen-bond geometry (Å, °)

*D*—H⋯*A*	*D*—H	H⋯*A*	*D*⋯*A*	*D*—H⋯*A*
O1—H1*A*⋯N1	0.82	1.92	2.639 (3)	146
C3—H3⋯*Cg*1^i^	0.93	2.76	3.661 (3)	164

Symmetry code: (i) 
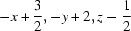
. *Cg* is the centroid of the C15–C20 benzene ring

**Fig. 1.**
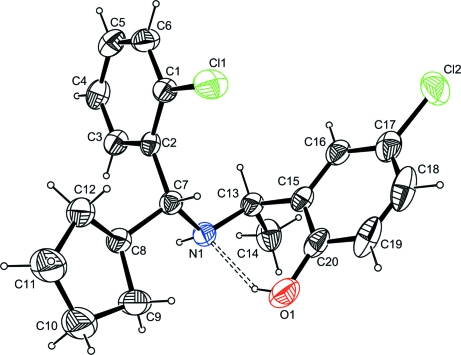
Molecular view of (I) with the atom-labeling scheme. Displacement ellipsoids are drawn at the 30% probability level. H atoms are shown as small spheres of arbitrary radii. H bond is shown as dashed line.

## supplementary crystallographic information

### Comment

The synthesis of enantiopure amine alcohols with a variety of functionalities is an important subject of research because this class of compounds has found widespread application in biological systems showing pharmacological activity. These compounds are used as resolving agents, chiral bases and auxiliaries in asymmetric synthesis (Cimarelli *et al.*,2002), and most have been derived from new readily available natural products (Ager *et al.*,1996). Chiral aminophenols, which are similar to aminoalcohols, are important building blocks in organic synthesis, and have attracted increasing attention in recent years owing to their effects in asymmetric synthesis and asymmetric induction (Cimarelli *et al.*,2001; Palmieri, 1999, 2000; Xu & Pu, 2004; Berrisford *et al.*, 1995; Cimarelli & Palmieri, 1998; Hayase *et al.*, 1997; Nakano *et al.*,1997; Soai *et al.*, 1992; Watanabe *et al.*, 1991).

As part of our continuing studies of chiral aminophenols, we have established the molecular structure of the title compound which was intially synthesized to test its asymmetric catalytic activity. The compound has been prepared by conventional condensation of (*R*)-1-(2-chlorophenyl)-1-cyclopentylmethanamine with 1-(5-chloro-2-hydroxyphenyl)ethanone, resulting in the formation of a new chiral center as shown in Fig. 1.

The structural analyses confirms the absolute configuration of the title compound and the formation of the (*R,R*) diastereoisomer. There is an intramolecular O-H···N hydrogen bond which stabilizes the conformation of the molecule. The molecules are linked to each other through weak C-H···π interaction involving the C15-C20 benzene ring (Table 1).

### Experimental

The title compound were prepared according to the procedure of Yang *et al.*(2005). (*R*)-1-(2-chlorophenyl)-1-cyclopentylmethanamine (0.9 mmol) and 1-(5-chloro-2-hydroxyphenyl)ethanone (0.9 mmol) were dissolved in methanol (10 ml) and reacted at room temperature for 48 h. After removal of the solvent, NaBH_4_ (4.5 mmol) was added to the solution in THF/ethanol (1:1 *v*/*v*, 20 ml) and stirred at 273 K until the solution became colourless. The solvent was then removed under reduced pressure. Water (10 ml)was added to the residue and 1 N HCl was added dropwise until hydrogen production ceased. The mixture was neutralized with aqueous Na_2_CO_3 _, then extracted with CHCl_3, _and the organic layer was dried over anhydrous sodium sulfate. The solvent was removed under reduced pressure. Further purification was carried out by thin-layer silica-gel chromatography (chloroform) to give a colourless solid (yield 83.5%). Crystals of (I) were grown from a n-hexane.

### Refinement

All H atoms were included in calculated positions and treated as riding on their parent atoms, with N—H = 0.90 Å, O—H = 0.82 Å, aromatic C—H = 0.93 Å, methyl C—H =0.96 Å, methylene C—H =0.97 Å and methine C—H = 0.98 Å, and with *U*_iso_(H) = 1.2*U*_eq_(C,*N*,*O*)or 1.5*U*_eq_(methyl C).

### Crystal data

**Table:**

C_20_H_23_Cl_2_NO	*F*(000) = 768
*M**_r_* = 364.29	*D*_x_ = 1.261 Mg m^−^^3^
Orthorhombic, *P*2_1_2_1_2_1_	Mo *K*α radiation, λ = 0.71073 Å
Hall symbol: P 2ac 2ab	Cell parameters from 2608 reflections
*a* = 11.286 (2) Å	θ = 2.2–20.5°
*b* = 11.539 (2) Å	µ = 0.34 mm^−^^1^
*c* = 14.740 (3) Å	*T* = 298 K
*V* = 1919.5 (6) Å^3^	Block, colourless
*Z* = 4	0.42 × 0.29 × 0.18 mm

### Data collection

**Table:**

Bruker SMART CCD area-detector diffractometer	3573 independent reflections
Radiation source: fine-focus sealed tube	2761 reflections with *I* > 2σ(*I*)
graphite	*R*_int_ = 0.032
φ and ω scans	θ_max_ = 25.5°, θ_min_ = 2.2°
Absorption correction: multi-scan (SADABS; Bruker, 1997)	*h* = −13→13
*T*_min_ = 0.869, *T*_max_ = 0.941	*k* = −13→11
10145 measured reflections	*l* = −17→15

### Refinement

**Table:**

Refinement on *F*^2^	Secondary atom site location: difference Fourier map
Least-squares matrix: full	Hydrogen site location: inferred from neighbouring sites
*R*[*F*^2^ > 2σ(*F*^2^)] = 0.048	H-atom parameters constrained
*wR*(*F*^2^) = 0.113	*w* = 1/[σ^2^(*F*_o_^2^) + (0.0486*P*)^2^ + 0.3378*P*] where *P* = (*F*_o_^2^ + 2*F*_c_^2^)/3
*S* = 1.02	(Δ/σ)_max_ < 0.001
3573 reflections	Δρ_max_ = 0.17 e Å^−^^3^
219 parameters	Δρ_min_ = −0.22 e Å^−^^3^
0 restraints	Absolute structure: Flack (1983), 1629 Friedel pairs
Primary atom site location: structure-invariant direct methods	Flack parameter: 0.03 (8)

### Special details

**Table:**

Geometry. All e.s.d.'s (except the e.s.d. in the dihedral angle between two l.s. planes) are estimated using the full covariance matrix. The cell e.s.d.'s are taken into account individually in the estimation of e.s.d.'s in distances, angles and torsion angles; correlations between e.s.d.'s in cell parameters are only used when they are defined by crystal symmetry. An approximate (isotropic) treatment of cell e.s.d.'s is used for estimating e.s.d.'s involving l.s. planes.
Refinement. Refinement of *F*^2^ against ALL reflections. The weighted *R*-factor *wR* and goodness of fit *S* are based on *F*^2^, conventional *R*-factors *R* are based on *F*, with *F* set to zero for negative *F*^2^. The threshold expression of *F*^2^ > σ(*F*^2^) is used only for calculating *R*-factors(gt) *etc*. and is not relevant to the choice of reflections for refinement. *R*-factors based on *F*^2^ are statistically about twice as large as those based on *F*, and *R*- factors based on ALL data will be even larger.

### Fractional atomic coordinates and isotropic or equivalent isotropic displacement parameters (Å^2^)

**Table:**

	*x*	*y*	*z*	*U*_iso_*/*U*_eq_	
C1	0.4448 (2)	0.9053 (2)	0.71545 (19)	0.0527 (7)	
C2	0.5587 (2)	0.9243 (2)	0.68485 (16)	0.0450 (6)	
C3	0.5716 (3)	1.0086 (2)	0.61779 (19)	0.0583 (7)	
H3	0.6468	1.0242	0.5952	0.070*	
C4	0.4774 (3)	1.0688 (3)	0.5843 (2)	0.0662 (9)	
H4	0.4891	1.1245	0.5395	0.079*	
C5	0.3652 (3)	1.0476 (3)	0.6164 (2)	0.0702 (9)	
H5	0.3007	1.0886	0.5939	0.084*	
C6	0.3501 (3)	0.9649 (3)	0.6822 (2)	0.0679 (9)	
H6	0.2746	0.9494	0.7043	0.082*	
C7	0.6676 (2)	0.8606 (2)	0.71942 (18)	0.0494 (7)	
H7	0.6412	0.8068	0.7667	0.059*	
C8	0.7239 (3)	0.7890 (3)	0.6445 (2)	0.0597 (8)	
H8	0.7496	0.8417	0.5963	0.072*	
C9	0.8298 (3)	0.7153 (3)	0.6731 (3)	0.0842 (11)	
H9A	0.9015	0.7616	0.6749	0.101*	
H9B	0.8168	0.6814	0.7324	0.101*	
C10	0.8389 (4)	0.6226 (4)	0.6016 (3)	0.1137 (16)	
H10A	0.8922	0.6467	0.5536	0.136*	
H10B	0.8687	0.5512	0.6278	0.136*	
C11	0.7165 (4)	0.6053 (4)	0.5653 (3)	0.1091 (15)	
H11A	0.7171	0.6091	0.4995	0.131*	
H11B	0.6863	0.5301	0.5834	0.131*	
C12	0.6404 (3)	0.6996 (3)	0.6033 (2)	0.0747 (10)	
H12A	0.5877	0.6688	0.6493	0.090*	
H12B	0.5930	0.7345	0.5557	0.090*	
C13	0.7159 (3)	1.0138 (3)	0.83381 (19)	0.0558 (7)	
H13	0.6433	1.0529	0.8140	0.067*	
C14	0.8106 (4)	1.1051 (3)	0.8523 (2)	0.0835 (11)	
H14A	0.8828	1.0677	0.8705	0.125*	
H14B	0.7842	1.1557	0.8999	0.125*	
H14C	0.8245	1.1494	0.7983	0.125*	
C15	0.6889 (3)	0.9446 (3)	0.91861 (19)	0.0537 (7)	
C16	0.6008 (3)	0.9804 (3)	0.9767 (2)	0.0606 (8)	
H16	0.5558	1.0452	0.9620	0.073*	
C17	0.5781 (3)	0.9215 (4)	1.0566 (2)	0.0757 (10)	
C18	0.6416 (5)	0.8248 (4)	1.0777 (3)	0.0969 (15)	
H18	0.6254	0.7844	1.1308	0.116*	
C19	0.7279 (4)	0.7875 (3)	1.0216 (3)	0.0909 (14)	
H19	0.7700	0.7207	1.0362	0.109*	
C20	0.7551 (3)	0.8472 (3)	0.9425 (2)	0.0669 (9)	
Cl1	0.41737 (8)	0.79949 (9)	0.79694 (7)	0.0876 (3)	
Cl2	0.47184 (9)	0.97355 (14)	1.13076 (7)	0.1212 (5)	
N1	0.75723 (19)	0.9378 (2)	0.76056 (16)	0.0590 (6)	
O1	0.8465 (2)	0.8094 (2)	0.89205 (18)	0.0974 (9)	
H1A	0.8471	0.8437	0.8433	0.146*	
H1	0.7853	0.9784	0.7146	0.117*	

### Atomic displacement parameters (Å^2^)

**Table:**

	*U*^11^	*U*^22^	*U*^33^	*U*^12^	*U*^13^	*U*^23^
C1	0.0501 (16)	0.0547 (17)	0.0533 (16)	−0.0074 (14)	0.0004 (14)	0.0055 (13)
C2	0.0518 (15)	0.0445 (14)	0.0386 (14)	−0.0076 (13)	−0.0041 (12)	−0.0018 (12)
C3	0.0571 (17)	0.0609 (18)	0.0567 (17)	−0.0135 (15)	−0.0047 (15)	0.0086 (15)
C4	0.088 (2)	0.0532 (19)	0.0577 (19)	−0.0095 (18)	−0.0138 (18)	0.0100 (15)
C5	0.074 (2)	0.063 (2)	0.074 (2)	0.0047 (18)	−0.0227 (19)	0.0004 (19)
C6	0.0472 (15)	0.078 (2)	0.079 (2)	−0.0036 (16)	−0.0035 (15)	0.0018 (19)
C7	0.0468 (15)	0.0528 (16)	0.0485 (16)	−0.0063 (13)	−0.0026 (13)	0.0001 (13)
C8	0.0586 (17)	0.0647 (19)	0.0558 (18)	−0.0008 (15)	−0.0021 (15)	−0.0062 (15)
C9	0.0568 (19)	0.093 (3)	0.102 (3)	0.013 (2)	−0.0081 (18)	−0.028 (2)
C10	0.088 (3)	0.124 (3)	0.129 (4)	0.024 (3)	−0.010 (3)	−0.059 (3)
C11	0.098 (3)	0.097 (3)	0.132 (4)	0.014 (3)	−0.008 (3)	−0.052 (3)
C12	0.068 (2)	0.077 (2)	0.079 (2)	0.0014 (19)	−0.0103 (18)	−0.0250 (19)
C13	0.0533 (16)	0.0598 (17)	0.0544 (17)	−0.0024 (15)	−0.0074 (14)	−0.0045 (14)
C14	0.099 (3)	0.082 (2)	0.069 (2)	−0.028 (2)	−0.001 (2)	−0.0123 (19)
C15	0.0525 (16)	0.0569 (17)	0.0516 (16)	−0.0085 (15)	−0.0162 (14)	−0.0034 (14)
C16	0.0534 (17)	0.070 (2)	0.0587 (19)	−0.0094 (16)	−0.0122 (15)	0.0018 (16)
C17	0.070 (2)	0.095 (3)	0.062 (2)	−0.035 (2)	−0.0145 (18)	0.000 (2)
C18	0.145 (4)	0.086 (3)	0.060 (2)	−0.051 (3)	−0.037 (3)	0.013 (2)
C19	0.143 (4)	0.059 (2)	0.071 (3)	−0.007 (2)	−0.057 (3)	0.001 (2)
C20	0.081 (2)	0.059 (2)	0.060 (2)	0.0069 (18)	−0.0332 (18)	−0.0136 (16)
Cl1	0.0671 (5)	0.1041 (7)	0.0916 (6)	−0.0106 (5)	0.0140 (5)	0.0452 (5)
Cl2	0.0834 (6)	0.2043 (14)	0.0759 (6)	−0.0444 (8)	0.0151 (5)	−0.0046 (8)
N1	0.0475 (13)	0.0749 (17)	0.0547 (14)	−0.0100 (13)	−0.0024 (11)	−0.0098 (13)
O1	0.1023 (19)	0.102 (2)	0.0875 (18)	0.0454 (17)	−0.0382 (15)	−0.0257 (16)

### Geometric parameters (Å, °)

**Table:**

C1—C6	1.363 (4)	C11—H11A	0.9700
C1—C2	1.379 (4)	C11—H11B	0.9700
C1—Cl1	1.740 (3)	C12—H12A	0.9700
C2—C3	1.394 (4)	C12—H12B	0.9700
C2—C7	1.520 (4)	C13—N1	1.468 (3)
C3—C4	1.363 (4)	C13—C15	1.515 (4)
C3—H3	0.9300	C13—C14	1.525 (4)
C4—C5	1.373 (5)	C13—H13	0.9800
C4—H4	0.9300	C14—H14A	0.9600
C5—C6	1.371 (5)	C14—H14B	0.9600
C5—H5	0.9300	C14—H14C	0.9600
C6—H6	0.9300	C15—C16	1.375 (4)
C7—N1	1.477 (3)	C15—C20	1.394 (4)
C7—C8	1.518 (4)	C16—C17	1.384 (4)
C7—H7	0.9800	C16—H16	0.9300
C8—C12	1.524 (4)	C17—C18	1.363 (6)
C8—C9	1.526 (4)	C17—Cl2	1.730 (4)
C8—H8	0.9800	C18—C19	1.348 (6)
C9—C10	1.504 (5)	C18—H18	0.9300
C9—H9A	0.9700	C19—C20	1.389 (5)
C9—H9B	0.9700	C19—H19	0.9300
C10—C11	1.495 (6)	C20—O1	1.344 (4)
C10—H10A	0.9700	N1—H1	0.8826
C10—H10B	0.9700	O1—H1A	0.8200
C11—C12	1.495 (5)		
			
C6—C1—C2	122.2 (3)	C10—C11—H11A	110.2
C6—C1—Cl1	117.6 (2)	C12—C11—H11B	110.2
C2—C1—Cl1	120.2 (2)	C10—C11—H11B	110.2
C1—C2—C3	116.1 (3)	H11A—C11—H11B	108.5
C1—C2—C7	124.5 (2)	C11—C12—C8	106.7 (3)
C3—C2—C7	119.4 (2)	C11—C12—H12A	110.4
C4—C3—C2	122.1 (3)	C8—C12—H12A	110.4
C4—C3—H3	118.9	C11—C12—H12B	110.4
C2—C3—H3	118.9	C8—C12—H12B	110.4
C3—C4—C5	120.2 (3)	H12A—C12—H12B	108.6
C3—C4—H4	119.9	N1—C13—C15	110.8 (2)
C5—C4—H4	119.9	N1—C13—C14	108.8 (2)
C6—C5—C4	118.9 (3)	C15—C13—C14	110.9 (2)
C6—C5—H5	120.6	N1—C13—H13	108.7
C4—C5—H5	120.6	C15—C13—H13	108.7
C1—C6—C5	120.5 (3)	C14—C13—H13	108.7
C1—C6—H6	119.7	C13—C14—H14A	109.5
C5—C6—H6	119.7	C13—C14—H14B	109.5
N1—C7—C8	109.9 (2)	H14A—C14—H14B	109.5
N1—C7—C2	113.6 (2)	C13—C14—H14C	109.5
C8—C7—C2	111.0 (2)	H14A—C14—H14C	109.5
N1—C7—H7	107.4	H14B—C14—H14C	109.5
C8—C7—H7	107.4	C16—C15—C20	118.2 (3)
C2—C7—H7	107.4	C16—C15—C13	120.0 (3)
C7—C8—C12	113.6 (2)	C20—C15—C13	121.7 (3)
C7—C8—C9	115.5 (3)	C15—C16—C17	121.1 (3)
C12—C8—C9	102.5 (3)	C15—C16—H16	119.4
C7—C8—H8	108.3	C17—C16—H16	119.4
C12—C8—H8	108.3	C18—C17—C16	119.9 (4)
C9—C8—H8	108.3	C18—C17—Cl2	120.3 (3)
C10—C9—C8	104.9 (3)	C16—C17—Cl2	119.8 (3)
C10—C9—H9A	110.8	C19—C18—C17	120.2 (4)
C8—C9—H9A	110.8	C19—C18—H18	119.9
C10—C9—H9B	110.8	C17—C18—H18	119.9
C8—C9—H9B	110.8	C18—C19—C20	121.1 (4)
H9A—C9—H9B	108.8	C18—C19—H19	119.5
C11—C10—C9	106.4 (3)	C20—C19—H19	119.5
C11—C10—H10A	110.4	O1—C20—C19	118.2 (3)
C9—C10—H10A	110.4	O1—C20—C15	122.2 (3)
C11—C10—H10B	110.4	C19—C20—C15	119.5 (4)
C9—C10—H10B	110.4	C13—N1—C7	116.4 (2)
H10A—C10—H10B	108.6	C13—N1—H1	111.2
C12—C11—C10	107.4 (3)	C7—N1—H1	104.5
C12—C11—H11A	110.2	C20—O1—H1A	109.5
			
C6—C1—C2—C3	0.2 (4)	C7—C8—C12—C11	−154.8 (3)
Cl1—C1—C2—C3	178.3 (2)	C9—C8—C12—C11	−29.4 (4)
C6—C1—C2—C7	179.9 (3)	N1—C13—C15—C16	−148.4 (2)
Cl1—C1—C2—C7	−1.9 (4)	C14—C13—C15—C16	90.6 (3)
C1—C2—C3—C4	0.0 (4)	N1—C13—C15—C20	34.8 (4)
C7—C2—C3—C4	−179.8 (3)	C14—C13—C15—C20	−86.2 (3)
C2—C3—C4—C5	0.0 (5)	C20—C15—C16—C17	−0.1 (4)
C3—C4—C5—C6	−0.2 (5)	C13—C15—C16—C17	−177.1 (3)
C2—C1—C6—C5	−0.3 (5)	C15—C16—C17—C18	−1.6 (5)
Cl1—C1—C6—C5	−178.5 (2)	C15—C16—C17—Cl2	176.6 (2)
C4—C5—C6—C1	0.3 (5)	C16—C17—C18—C19	1.2 (5)
C1—C2—C7—N1	−119.6 (3)	Cl2—C17—C18—C19	−177.0 (3)
C3—C2—C7—N1	60.2 (3)	C17—C18—C19—C20	1.0 (6)
C1—C2—C7—C8	116.1 (3)	C18—C19—C20—O1	176.3 (3)
C3—C2—C7—C8	−64.2 (3)	C18—C19—C20—C15	−2.7 (5)
N1—C7—C8—C12	175.2 (3)	C16—C15—C20—O1	−176.8 (3)
C2—C7—C8—C12	−58.3 (3)	C13—C15—C20—O1	0.1 (4)
N1—C7—C8—C9	57.2 (3)	C16—C15—C20—C19	2.2 (4)
C2—C7—C8—C9	−176.4 (3)	C13—C15—C20—C19	179.1 (3)
C7—C8—C9—C10	158.9 (3)	C15—C13—N1—C7	70.8 (3)
C12—C8—C9—C10	34.8 (4)	C14—C13—N1—C7	−166.9 (3)
C8—C9—C10—C11	−27.7 (5)	C8—C7—N1—C13	179.4 (2)
C9—C10—C11—C12	9.1 (5)	C2—C7—N1—C13	54.4 (3)
C10—C11—C12—C8	13.0 (5)		

### Hydrogen-bond geometry (Å, °)

**Table:**

*D*—H···*A*	*D*—H	H···*A*	*D*···*A*	*D*—H···*A*
O1—H1A···N1	0.82	1.92	2.639 (3)	146
C3—H3···Cg1^i^	0.93	2.76	3.661 (3)	164

Symmetry codes: (i) −*x*+3/2, −*y*+2, *z*−1/2.
